# Complete mitochondrial genome of *Glomeridesmus spelaeus* (Diplopoda, Glomeridesmida), a troglobitic species from iron-ore caves in Eastern Amazon

**DOI:** 10.1080/23802359.2020.1812450

**Published:** 2020-08-31

**Authors:** Gisele Lopes Nunes, Renato Renison Moreira Oliveira, Eder Soares Pires, Thadeu Pietrobon, Xavier Prous, Guilherme Oliveira, Santelmo Vasconcelos

**Affiliations:** a Instituto Tecnológico Vale, Belém, Brazil; b Speleology, Vale S.A., Nova Lima, Brazil

**Keywords:** Millipede, mitogenome, Serra dos Carajás, troglobite

## Abstract

We report the complete mitochondrial genome sequence of *Glomeridesmus spelaeus*, the first sequenced genome of the order Gomeridesmida. The genome is 14,825 pb in length and encodes 37 mitochondrial (13 PCGs, 2 rRNA genes, 22 tRNA) genes and contains a typical AT-rich region. The base composition of the mitogenome was A (40.1%), T (36.4%), C (15.8%), and G (7.6%), with an GC content of 23.5%. Our results indicated that *G. spelaeus* is only distantly related to the other Diplopoda species with available mitochondrial genomes in the public databases. As the broadest genetic characterization of a Glomeridesmida species available to date, the mitogenome of *G. spelaeus* will help understanding the evolution of such a little-known millipede group. Also, our data will be important for the characterization and conservation of the diverse invertebrate troglofauna of the Amazonian caves.

There are a large number of caves in Brazil, although only 7% are registered of a total estimated of 100,000 caves (Sallun Filho and Karmann 2012; Auler and Piló 2015). These registers are temporally validated, based in environmental studies conducted for mining operations, by National Center for Research and Conservation of Caves (http://www.icmbio.gov.br/cecav). The Brazilian legislation considers caves as special environmental entities subjected to specific protective measures that are based on the levels of relevance from maximum to minimum (Auler and Piló 2015). Relevance levels depend both on physical and biological attributes, among others, although the last are significantly more important in the evaluation process of the caves, thus highlighting the central importance of studies on the cave biota (Jaffé et al. 2016). Nevertheless, there is a large knowledge gap on the identification of the cave fauna, especially troglobites, i.e. those inhabiting exclusively the cave environment.

*Glomeridesmus spelaeus* is a troglobite species of Diplopoda found in iron ore caves in the Carajás region in the Amazon basin (Iniesta et al. 2012). *Glomeridesmus* are chilognath millipedes belonging to Glomeridesmida, a small order composed of 31 species (26 Glomeridesmidae and five Termitodesmidae) (Jeekel 2003), which may be basal to pill millipedes and other groups of Diplopoda (Hoffman 1982). In addition, very little is known on the troglobitic Glomeridesmida, with only two records described so far: *G. sbordonii* (Shear 1974) from Grutas de Cocona (Teapa, Tabasco, Mexico) and *G. spelaeus* (Iniesta et al. 2012) from iron-ore caves in Serra dos Carajás, Eastern Amazon (Curionópolis, Pará, Brazil). Therefore, by describing the mitogenome of the troglobite *G. spelaeus*, we aimed to provide a robust genetic characterization to contribute to a more comprehensive understanding of one of the least known arthropod orders.

One specimen of *G. spelaeus* was collected in an iron-ore cave (6°04′52″S, 50°08′03″W) in Serra dos Carajás, Pará, Brazil. Total genomic DNA was extracted using DNeasy Blood & Tissue Kit (Qiagen), following the recommended protocol for insects. Paired-end libraries (2 × 75 bp) were built using QXT Sure Select (Agillent Technologies) and sequenced on the NextSeq 500 platform (Illumina), generating 29,527,776 high-quality reads. NOVOPlasty 2.6.3 (Dierckxsens et al. 2017) and Geneious Prime (Biomatters) were used for a *de novo* assembly of the mitogenome, resulting in a circular mitochondrial genome. Annotations were carried out with the MITOS (Bernt et al. 2013).

The mitogenome of *G. spelaeus* (GenBank accession no. MG372113) presented 14,825 bp, including 13 protein-coding genes (PCGs), 2 ribosomal RNAs, and 22 transfer RNAs. The overall nucleotide composition of the mitogenome was 40.1% of A, 15.8% of C, 7.6% of G, and 36.4% of T. The AT and GC contents were 76.5% and 23.5%, respectively. We observed most genes encoded on the H-strand, except for NAD1, NAD4, NAD4L, NAD5, trnC, trnF, trnH, trnI, trnL1(tag), trnL2(taa), trnP, trnQ, trnV and trnY, which were encoded on the L-strand. The genes that were not initiated with ATG codon, started with ATA (COX1, NAD4L, CYTB) and ATT (NAD1, NAD2, NAD3, NAD5, NAD6 and ATP8). All PCG sequences terminated with the conventional stop codon TAA.

We performed a phylogenetic analysis based on all mtDNA genes from all available mitochondrial genomes of Diplopoda species in GenBank (*Abacion magnum*, GenBank no. JX437062; *Anaulaciulus koreanus*, KX096886; *Antrokoreana gracilipes*, DQ344025; *Appalachioria falcifera*, JX437063; *Asiomorpha coarctata*, KU721885; *Brachycybe lecontii*, JX437064; Sphaerotheriidae sp., JQ713564; and *Xystodesmus* sp., KU721886), besides and two Chilopoda mitogenomes as outgroup (*Cermatobius longicornis*, KC155628; and *Scutigera coleoptrata*, AJ507061) (Figure 1). All used genes were aligned separately in MAFFT 7.3 (Katoh and Standley 2013), using the algorithm *Auto*, and then concatenated in a single matrix. Afterwards, the phylogenetic tree was obtained through the maximum likelihood approach implemented in RAxML 8.2 (Stamatakis 2014), using the GTR + G model and rapid bootstraping with 1000 replicates (Figure 1).

**Figure 1.**
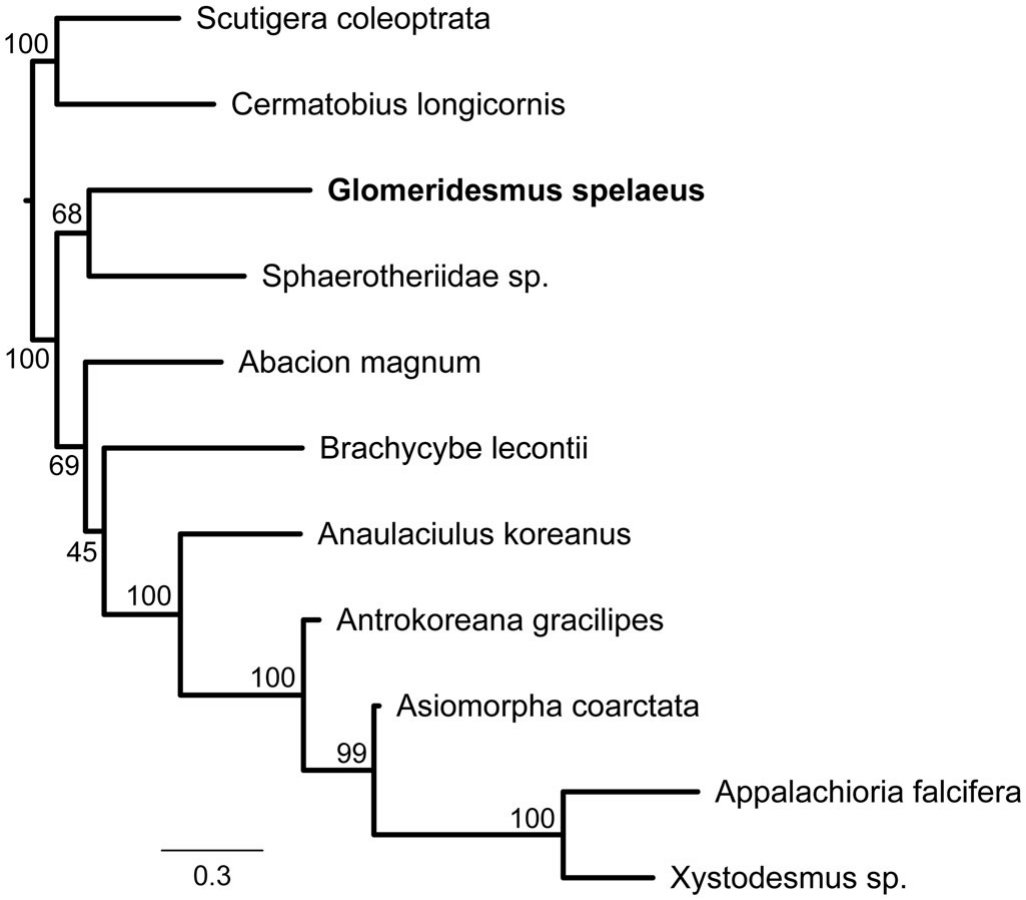
Maximum likelihood major-rule consensus tree showing the phylogenetic placement tree of *Glomeridesmus spelaeus* among Diplopoda species with available mitogenomes in GenBank. Bootstrap support values are shown near the clade branches.

The phylogenetic tree evidenced generally high bootstrap values (BS ≥ 99%), with *G. spelaeus* as only distantly related to the other Diplopoda species with available mitochondrial genomes in public databases, thus emphasizing the remarkable genetic information gap not only for the order Glomeridesmida, but also for millipedes in general. Research including troglobitic species are essential to understand both speciation and evolution processes of such unique organisms that live exclusively in caves. Genetic data on troglobitic species will be crucial for the determination of the biodiversity present in the ferruginous caves of Serra dos Carajás, besides contributing to the unraveling of the evolutionary patterns of the genus *Glomeridesmus*.

## Data Availability

The data that support the findings of this study are available in NCBI at https://www.ncbi.nlm.nih.gov, reference number MG372113.
